# Transcriptional interference by RNA polymerase III affects expression of the *Polr3e* gene

**DOI:** 10.1101/gad.293324.116

**Published:** 2017-02-15

**Authors:** Meghdad Yeganeh, Viviane Praz, Pascal Cousin, Nouria Hernandez

**Affiliations:** 1 Center for Integrative Genomics, Faculty of Biology and Medicine, University of Lausanne, 1015 Lausanne, Switzerland;; 2 Swiss Institute of Bioinformatics, 1015 Lausanne, Switzerland

**Keywords:** RNA polymerase, gene expression regulation, *Polr3e* gene, antisense transcription, transcriptional interference, SINE

## Abstract

Overlapping gene arrangements can potentially contribute to gene expression regulation. A mammalian interspersed repeat (MIR) nested in antisense orientation within the first intron of the *Polr3e* gene, encoding an RNA polymerase III (Pol III) subunit, is conserved in mammals and highly occupied by Pol III. Using a fluorescence assay, CRISPR/Cas9-mediated deletion of the MIR in mouse embryonic stem cells, and chromatin immunoprecipitation assays, we show that the MIR affects *Polr3e* expression through transcriptional interference. Our study reveals a mechanism by which a Pol II gene can be regulated at the transcription elongation level by transcription of an embedded antisense Pol III gene.

## Abstract (precis)

A mammalian interspersed repeat (MIR) nested in antisense orientation within the first intron of the *Polr3e* gene, encoding an RNA polymerase (Pol) III subunit, is conserved in mammals and highly occupied by Pol III. Here, Yeganeh et al. show that the MIR affects *Polr3e* expression through transcriptional interference.

In eukaryotes, RNA polymerase II (Pol II) is responsible for transcription of all of the mRNA-encoding genes as well as most genes encoding small nuclear RNA (snRNA) and microRNAs. Pol II-dependent transcription occurs in several steps, each of which can be subjected to regulation in response to environmental and genetic signaling processes (Fuda et al. 2009). One of the highly regulated steps is the transition from initiation to productive elongation, which is controlled by several positive and negative regulatory factors (Zhou et al. 2012). In particular, the 5,6-dichloro-1-β-d-ribofuranosylbenzimidazole (DRB) sensitivity-inducing factor (DSIF) and the negative elongation factor (NELF) cause the polymerase to pause just downstream from the transcription start site (TSS). Entering productive elongation involves DSIF and NELF phosphorylation and loss of NELF from the transcription complex. Once the polymerase has entered productive elongation, its elongation rate is highly dynamic and varies across a gene, in particular to allow cotranscriptional events to be performed efficiently. The largest Pol II accumulations detected by chromatin immunoprecipitation (ChIP) assays typically correspond to promoter-proximal pausing before productive elongation and slowing down near the gene 3′ end for cotranscriptional polyadenylation of the transcript (Jonkers and Lis 2015). The distribution of DSIF follows a similar pattern, whereas NELF is typically found only at the promoter-proximal pause region (Zhou et al. 2012).

Genes often lie in overlapping arrangements on either the same strand or opposite strands. The overlap can be partial, or an entire gene may be located or “nested” inside another gene, usually within an intron (Kumar 2009). A frequent arrangement found in both yeast and mammals but studied mostly in yeast is a long noncoding RNA (lncRNA; natural antisense transcript) gene present in antisense orientation relative to a protein-coding gene (Katayama et al. 2005; Huber et al. 2016). The different arrangements of overlapping genes can contribute to the regulation of gene expression by a number of mechanisms involving, in general, the natural antisense transcripts and/or, in some cases, the process of overlapping transcription (Pelechano and Steinmetz 2013). As recent examples, in yeast, a *CDC28* antisense lncRNA induced upon osmotic stress mediates gene looping and the transfer of Hog1 and associated factors from the 3′ untranslated region (UTR) to the *CDC28* TSS region, resulting in *CDC28* transcription activation (Nadal-Ribelles et al. 2014). In mammalian cells, the lncRNA Wrap53, an antisense transcript originating from the *p53* locus, binds CTCF and contributes to p53 regulation (Saldana-Meyer et al. 2014), and as a third example, a lncRNA antisense to the *Nos1* locus down-regulates Nos1 protein but not *Nos1* mRNA, suggesting a post-transcriptional effect (Korneev et al. 2015).

Most cases of gene regulation by an overlapping ncRNA gene in eukaryotes concern arrangements in which both genes are transcribed by Pol II, with very few studied examples of Pol II–Pol I and Pol II–Pol III gene overlaps. Among these, the Pol II TAR1 gene is nested within the Pol I 25S rRNA gene in *Saccharomyces cerevisiae,* but the influence, if any, of this layout on gene expression has not been examined directly (Coelho et al. 2002). In *Arabidopsis thaliana*, a set of nine Pol III proline transfer RNA (tRNA) genes is located antisense of the Pol II AtNUDT22 gene, and the two genes display negatively correlated expression levels, consistent with the possibility that they influence each other's expression (Lukoszek et al. 2013).

In addition to genes of known function, Pol III transcribes some short interspersed nuclear elements (SINEs). SINEs originated by retrotransposition mostly from tRNA and *Rn7sl* genes, often resulting in the transposed element carrying the gene-internal promoter of the source gene (Dieci et al. 2013). Although most SINEs are epigenetically repressed, some are actively transcribed by Pol III as independent transcription units (Roberts et al. 2003; Barski et al. 2010; Canella et al. 2010, 2012; Moqtaderi et al. 2010; Oler et al. 2010; Raha et al. 2010; Renaud et al. 2014). SINEs have long been considered as junk DNA, but it is now clear that they can profoundly impact genome functions both in *cis* (for example, by constituting new enhancers or splice sites) and in *trans* (for example, by producing RNAs that affect Pol II transcription) (Kramerov and Vassetzky 2011). Here we examined the role of a member of the mammalian interspersed repeat (MIR) family, an ancient family of tRNA-derived SINEs that were amplified before the mammalian radiation (Smit and Riggs 1995). This MIR is nested in antisense orientation within the first intron of the *Polr3e* gene, which codes for one of the Pol III subunits. We show that this arrangement is conserved in different mammalian species and that it directly impacts on Pol II transcription elongation through the *Polr3e* gene. Thus, the Pol III transcribed MIR can contribute to regulation of a Pol III subunit-encoding gene.

## Results

### A MIR in the first intron of the *Polr3e* gene is conserved among mammalian species and highly occupied by both Pol III and Pol II

**Figure 1.**
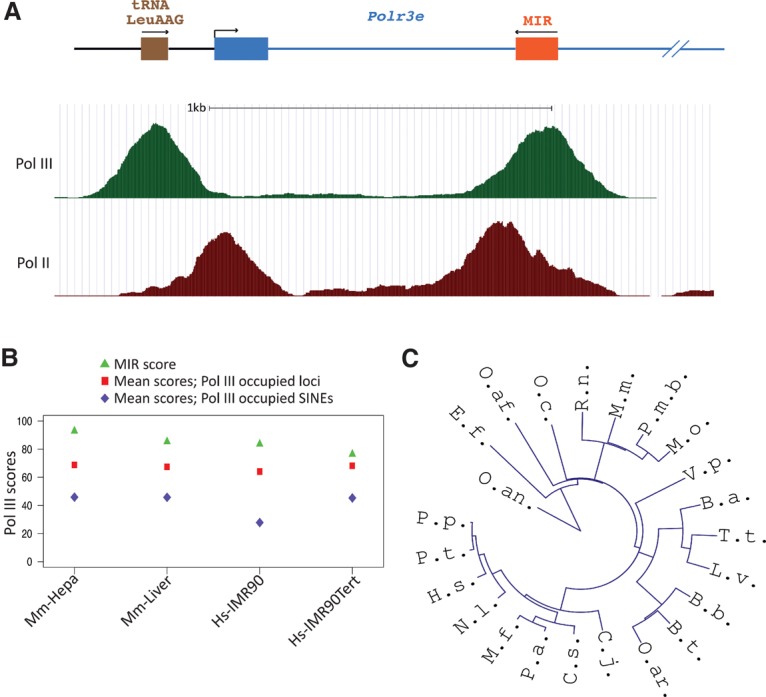
A MIR in the first intron of the *Polr3e* gene is conserved among mammals and highly occupied by Pol III and Pol II. (*A*) The genomic arrangement of the beginning of the mouse *Polr3e* gene is shown at the *top*. A tRNA Leu gene is located upstream of the *Polr3e* TSS. The MIR in the first intron is transcribed in the antisense direction. The *bottom* part shows a University of California at Santa Cruz (UCSC) genome browser view of Pol III and Pol II ChIP-seq profiles in mouse livers. (*B*) MIR, average Pol III-occupied locus, and average Pol III-occupied SINE Pol III occupancy scores [log_2_(immunoprecipitation/input)] shown as percentages in mouse hepa1–6 cells, mouse livers, IMR90 cells, and IMR90tert cells. (*C*) Alignment tree of MIR sequences in the first intron of *Polr3e* in different mammalian species, including *Balaenoptera acutorostrata* (B.a.), *Bos taurus* (B.t.), *Bubalus bubalis* (B.b.), *Callithrix jacchus* (C.j.), *Chlorocebus sabaeus* (C.s.), *Eptesicus fuscus* (E.f.), *Homo sapiens* (H.s.), *Lipotes vexillifer* (L.v.), *Macaca fascicularis* (M.f.), *Microtus ochrogaster* (M.o.), *Mus musculus* (M.m), *Nomascus leucogenys* (N.l.), *Ornithorhynchus anatinus* (O.an.), *Orycteropus afer* (O.af.), *Oryctolagus cuniculus* (O.c.), *Ovis aries* (O.ar.), *Pan paniscus* (P.p.), *Pan troglodytes* (P.t.), *Papio anubis* (P.a.), *Peromyscus maniculatus bairdii* (P.m.b), *Rattus norvegicus* (R.n.), *Tursiops truncates* (T.t), and *Vicugna pacos* (V.p.). The tree is based on the alignment in Supplemental Figure S1 and was generated from Jalview with the neighbor joining based on percent identity; the file was processed at the Interactive Tree of Life (iTOL) Web site (http://itol.embl.de) for circular graphic representation.

In both the mouse and human genomes, the first intron of the *Polr3e* gene contains an antisense MIR SINE (Canella et al. 2012). ChIP-seq (ChIP combined with high-throughput sequencing) data obtained from mouse livers reveal that this MIR is as highly occupied by Pol III as a tRNA Leu gene located upstream of the *Polr3e* TSS (Fig. 1A). Indeed, this MIR was found to be highly occupied by Pol III as compared with the mean occupancy scores of either all Pol III-occupied loci or just SINEs in not only mouse livers but also a mouse hepatocarcinoma cell line and human IMR90 and IMR90Tert cell lines (Fig. 1B; Renaud et al. 2014; Orioli et al. 2016), consistent with its high occupancy also in HeLa cells (Oler et al. 2010).

The high Pol III occupancy of this particular MIR is in contrast to the low occupancy of most SINEs and prompted us to search for its presence in other species. We found MIR-related sequences located antisense in the first intron of the *Polr3e* genes of all examined mammalian species, including the monotreme platypus (*Ornithorhynchus anatinus*), as illustrated by the sequence similarity tree in Figure 1C. The sequence alignment in Supplemental Figure S1 shows that all of these MIRs have potentially functional type 2 Pol III promoters; i.e., gene-internal A and B boxes separated by 25–26 base pairs (bp). This conservation is consistent with MIRs having amplified before the mammalian radiation (Smit and Riggs 1995) and suggests that the MIR in the first intron of the *Polr3e* gene might have a function.

**Figure 2.**
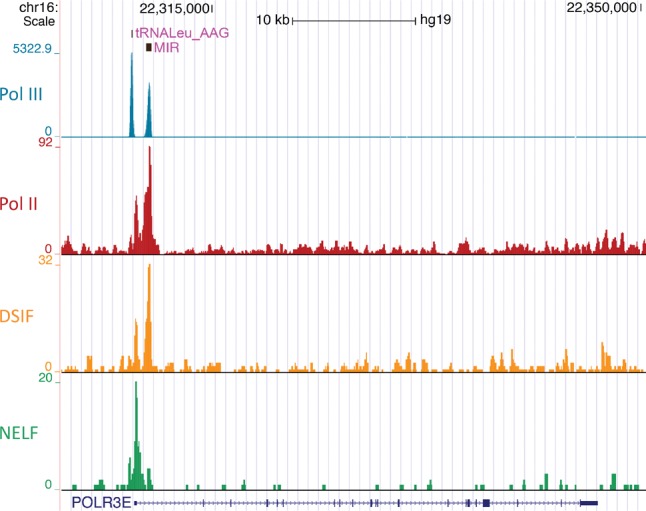
UCSC genome browser views of Pol III, Pol II, DSIF, and NELF ChIP-seq profiles in HeLa cells in the *POLR3E* gene region. Tracks are from ENCODE (Pol III subunit RPC1) and Liu et al. (2014) (Pol II, DSIF, and NELF).

When examining the Pol II and Pol III occupancy patterns in Figure 1A, we noticed a striking accumulation of Pol II not only at the TSS, as expected from pausing before escape into productive elongation, but also just before the antisense Pol III MIR (Fig. 1A; see also Canella et al. 2012). ChIP-seq data from HeLa cells (Liu et al. 2014) show DSIF accumulation near both the TSS and the MIR but NELF accumulation only near the TSS, arguing against Pol II accumulation at the MIR resulting from a second, unannotated TSS in this region (Fig. 2). A possible interpretation is that the MIR contributes to a Pol II accumulation at its 3′ end through either transcription interference or a *trans*-acting mechanism involving the MIR RNA.

### Active transcription of the MIR in antisense, but not sense, orientation within an EGFP-expressing construct leads to decreased fluorescence intensity

**Figure 3.**
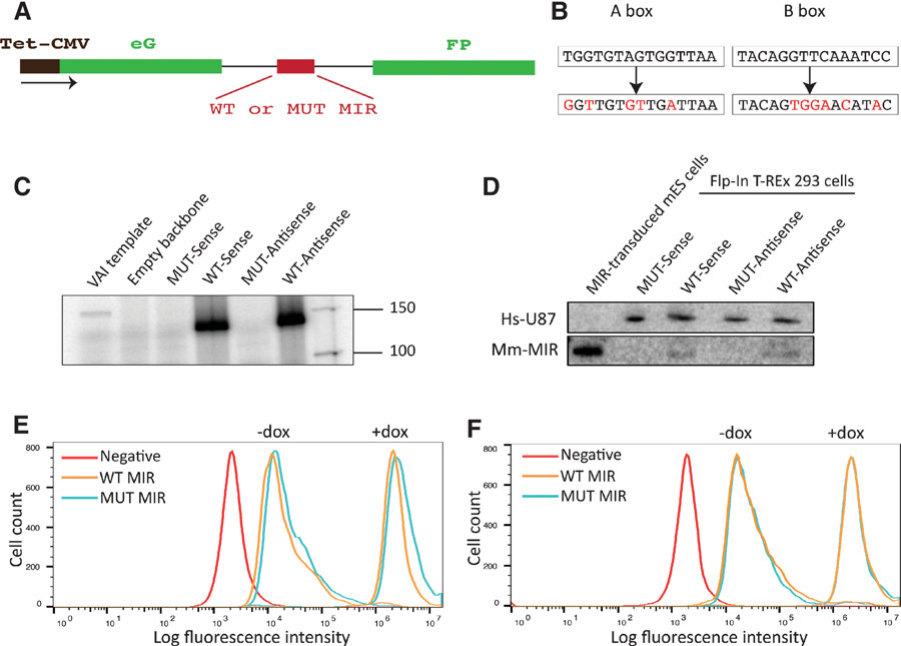
Active transcription of the MIR in antisense, but not sense, orientation within an EGFP-expressing construct leads to decreased fluorescence intensity. (*A*) Schematic view of the EGFP construct. EGFP-coding sequence under a tetracycline-inducible promoter is interrupted by an intron in which wild-type (WT) or mutant (MUT) MIR in sense or antisense orientation is inserted. (*B*) Mutations introduced in the A and B boxes of the MIR. Changes are indicated in red. (*C*) In vitro transcription performed with the templates indicated at the *top*: mutant MIR or wild-type MIR inserted in the EGFP intron in sense or antisense orientation. The Adenovirus 2 VAI gene was used as a positive control. (*D*) Northern blot analysis of mouse MIR expression in human Flp-In T-REx 293 cells transfected with the constructs indicated at the *top*. Total RNA extracted from mouse embryonic stem (ES) cells transduced with a construct containing the same MIR insert as in the EGFP constructs served as a positive control for MIR size. As an internal control for loading, the blot was probed with an oligonucleotide complementary to human U87. (*E*) FACS analysis of cells transfected with EGFP constructs containing wild-type and mutant MIR in antisense orientation represented as cell count (*Y*-axis) versus log fluorescence intensity (*X-*axis) either noninduced or induced with 2 µg/mL doxycycline and collected 72 h after induction. Nontransfected Flp-In T-REx 293 cells were used as a negative control. The experiment was repeated four times. *P*-value_−dox_ = 0.000023; *P*-value_+dox_ = 0.000016, calculated using Student's *t*-test. (*F*) As in *E* but with cells transfected with wild-type or mutant sense constructs. *P*-value_−dox_ = 0.131; *P*-value_+dox_ = 0.167.

To examine the effect of the MIR on expression of an overlapping Pol II gene, we placed the MIR (either wild type or with mutated A and B boxes in either sense or antisense orientation) within an intron inserted into the EGFP-coding sequence (Fig. 3A,B; Santillan et al. 2014). In vitro transcription assays with these constructs revealed robust and intact A-box- and B-box-dependent expression of both the sense and antisense MIR (Fig. 3C). We thus used these constructs to create stable inducible cell lines by cotransfection into Flp-In T-REx 293 cells along with a plasmid expressing Flp-recombinase and selection of the transfected cells with hygromycine. Northern blotting revealed weak but clearly detectable A-box- and B-box-dependent expression from both the sense and antisense MIRs (Fig. 3D). The relatively weak signal, which is in contrast to robust MIR expression in vitro, suggests rapid degradation of the MIR transcript in the cell.

We measured EGFP expression by FACS in either noninduced cells or cells induced for EGFP expression by doxycycline. When the MIR was antisense relative to EGFP transcription, fluorescence intensity was decreased slightly (20%–30%) but reproducibly in cells containing the wild-type MIR as compared with cells containing the mutant MIR construct (Fig. 3E). This was true at both the low leaky EGFP expression levels in the absence of doxycycline and at high doxycycline-induced EGFP expression levels. In contrast, no measurable effect was observed in this assay when the MIR was in the sense orientation (Fig. 3F). Thus, a Pol III transcribed MIR can reduce expression of a Pol II gene in which it is embedded in antisense orientation.

### CRISPR/Cas9-mediated deletion of the MIR leads to increased expression of *Polr3e*

**Figure 4.**
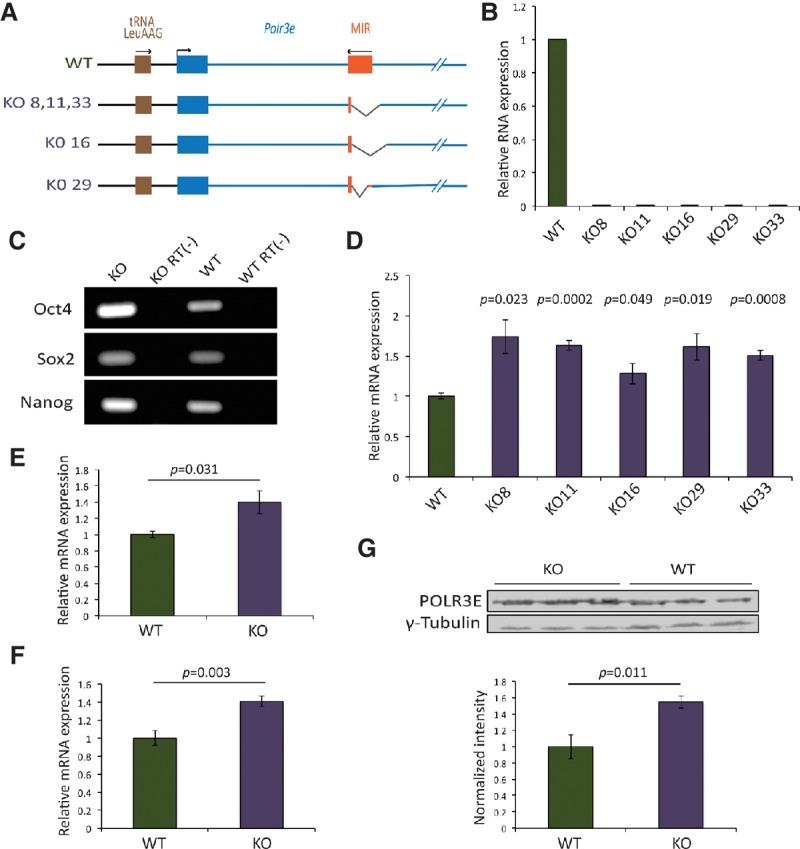
CRISPR/Cas9-mediated deletion of the MIR leads to increased expression of *Polr3e*. (*A*) Schematic view of CRISPR/Cas9-mediated deletions in different clones (#8, #11, #16, #29, and #33). All deletions maintain intact the region corresponding to the 3′ end of the MIR. (*B*) RT-qPCR performed with MIR-specific primers for both reverse transcription and qPCR with RNA extracted from the indicated cell lines. The qPCR results were normalized to *Gapdh* expression. (*C*) RT–PCR performed with total RNA from KO11 and wild-type ES cells to monitor expression of the pluripotency genes *Oct4*, *Sox2*, and *Nanog*. In the control RT(−) lanes, no reverse transcriptase was included in the reactions. (*D*) Results of RT-qPCR performed with primers detecting both pre-mRNA and mature *Polr3e* mRNA in the indicated ES knockout cell lines relative to wild-type cells. The qPCR results were normalized to *Actb* mRNA. Error bars represent ± SEM. *n* = 3. The *P*-values were calculated using Student's *t*-test and are relative to the wild type. (*E*) Results of RT-qPCR performed with primers detecting *Polr3e* pre-mRNA in the knockout ES cells relative to wild-type cells. The results were normalized to *Actb* expression. Error bars and *P*-values are as in *D*. (*F*) As in *E* but with primers detecting only mature *Polr3e* mRNA. (*G*, *top*) Western blot performed with anti-POLR3E and anti-γ-tubulin antibodies with protein extracts from knockout and wild-type ES cells. (*Bottom*) The POLR3E band intensities were quantified and normalized to their corresponding γ-tubulin band intensities. Error bars and *P*-values are as in *D*.

To determine the effect of the MIR in its natural genomic context, we used the CRISPR/Cas9 system to delete MIR genomic sequences in mouse embryonic stem (ES) cells. A schematic view of deletions obtained in different ES cell clones is shown in Figure 4A. We engineered deletions that left intact the 3′ region of the MIR where our ChIP-seq data had revealed accumulation of Pol II. As expected, MIR RNA was absent from these cell lines as determined by RT-qPCR (Fig. 4B), and at least the one cell line (KO11) that we tested still expressed the three pluripotency transcription factor-encoding genes *Oct4*, *Sox2*, and *Nanog* despite a number of cell passages imposed by genome engineering and single-cell cloning (Fig. 4C).

We then measured *Polr3e* mRNA expression levels in the various MIR knockout cells and wild-type cells by RT-qPCR with qPCR primers located inside a single exon toward the 3′ end of the *Polr3e* transcription unit (i.e., measuring both processed and unprocessed transcripts) and observed increased *Polr3e* total mRNA levels in all MIR knockout cell lines as compared with wild-type cells (Fig. 4D).

We focused on the MIR-deleted cell line KO11. To determine whether the increase in total mRNA level of *Polr3e* reflected increased transcription or any post-transcriptional effect such as increased stability, we measured levels of *Polr3e* pre-mRNA and mature mRNA in wild-type and KO11 cells by RT-qPCR. We designed (1) qPCR primers inside a single intron and (2) a primer at an exon–exon junction paired with another within an exon to amplify specifically precursor and mature mRNAs, respectively. We observed increased levels of both unspliced (Fig. 4E) and spliced (Fig. 4F) *Polr3e* mRNA in KO11 cells relative to wild-type cells, and this increase in turn resulted in an almost 1.5-fold increase in protein levels as determined by Western blot (Fig. 4G). However, these increased POLR3E levels did not lead to increased levels of 5S rRNA, pre-tRNA Ile, and U6 snRNA (Supplemental Fig. S2), suggesting that this Pol III subunit is not limiting for Pol III activity under the conditions tested.

**Figure 5.**
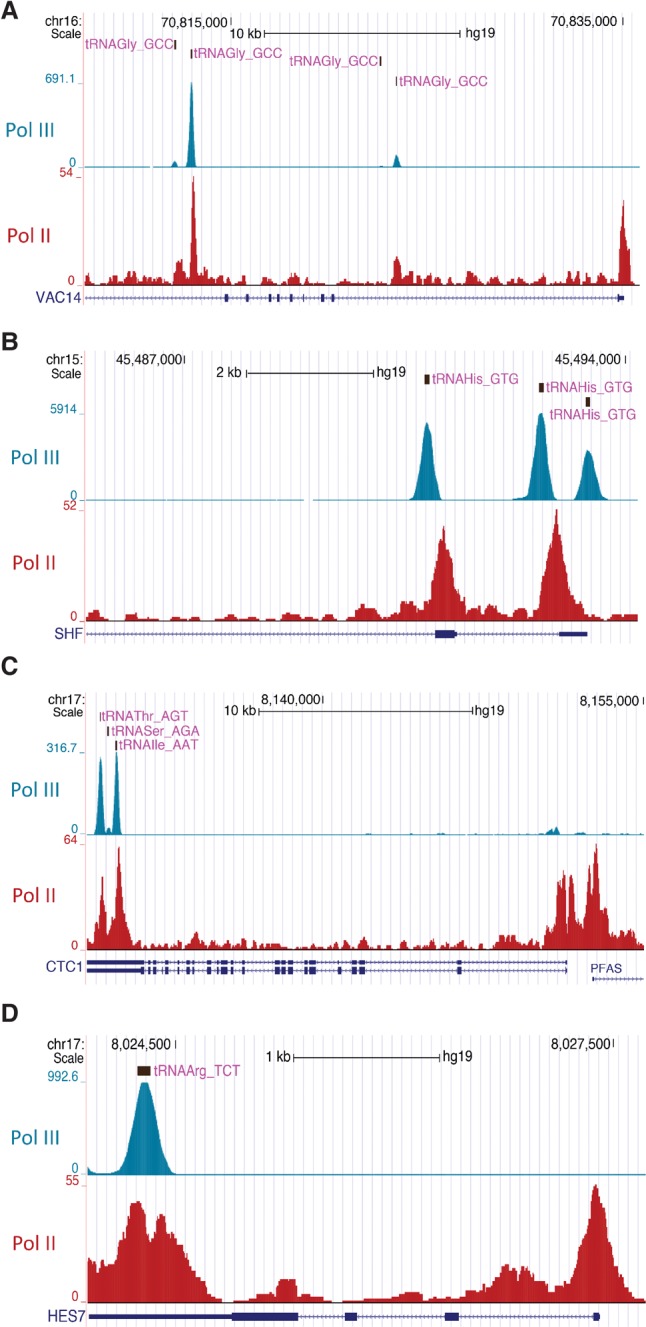
UCSC genome browser views showing examples of Pol II accumulations overlapping with Pol III peaks within the *VAC14* (*A*), *SHF* (*B*), *CTC1* (*C*), and *HES7* (*D*) genes. Tracks are from ENCODE (RPC1) and Liu et al. (2014) (Pol II).

The regulation of Pol II gene expression by a nested Pol III gene shown here suggests the existence of another layer of gene expression regulation by interplay between RNA polymerases. To determine how general this phenomenon might be, we examined all of the Pol III (RPC1) ENCODE peaks from HeLa cells and extracted those embedded in a Pol II transcription unit. We found 984 ENCODE Pol III peaks within Pol II genes, but examination of several of those RPC1 peaks in Pol II-occupied genes (for example, peaks located within the *CDA*, *KPNA6*, *YARS*, *STK40*, *CTH*, *USP33*, *DNAJB4*, and *SOAT1* Pol II transcription units on chromosome 1) did not reveal corresponding accumulations of Pol II. However, in the human genome, the analysis of anti-Pol III ChIP-seq experiments is complicated by the presence of a large number of repetitive sequences derived from Pol III transcription units; as a result, a large proportion of the sequence tags obtained in such experiments matches several locations (sometimes several hundred locations) in the genome. We therefore remapped the ENCODE sequence tags and recalculated peak scores using our previously described method (Canella et al. 2012), which assigns different weights to tags according to the number of times they were sequenced and the number of corresponding matches in the genome. The results confirmed 22 of the 984 peaks, of which 15 coincided with RPC4 peaks observed in IMR90Tert cells (see Supplemental Table S1; Orioli et al. 2016). These 15 Pol III peaks were located in nine different Pol II transcription units, five of which were clearly occupied by Pol II (Liu et al. 2014) and, importantly, clearly displayed Pol II accumulations at locations of Pol III peaks, as shown in Figure 5 for the *VAC14*, *SHF*, *CTC1*, and *HES7* genes. Such accumulations occurred when the Pol III transcription unit was orientated sense or antisense relative to the Pol II gene (Fig. 5). The results show that at least in HeLa and IMR90Tert cells, there are very few cases of Pol III-occupied transcription units leading to intragenic Pol II accumulations; they further suggest that both sense and antisense Pol III transcription units can lead to Pol II roadblocks, although the final effect on gene expression is likely to depend on both Pol II and Pol III transcription levels.

### The MIR effect on *Polr3e* is mediated by transcriptional interference

**Figure 6.**
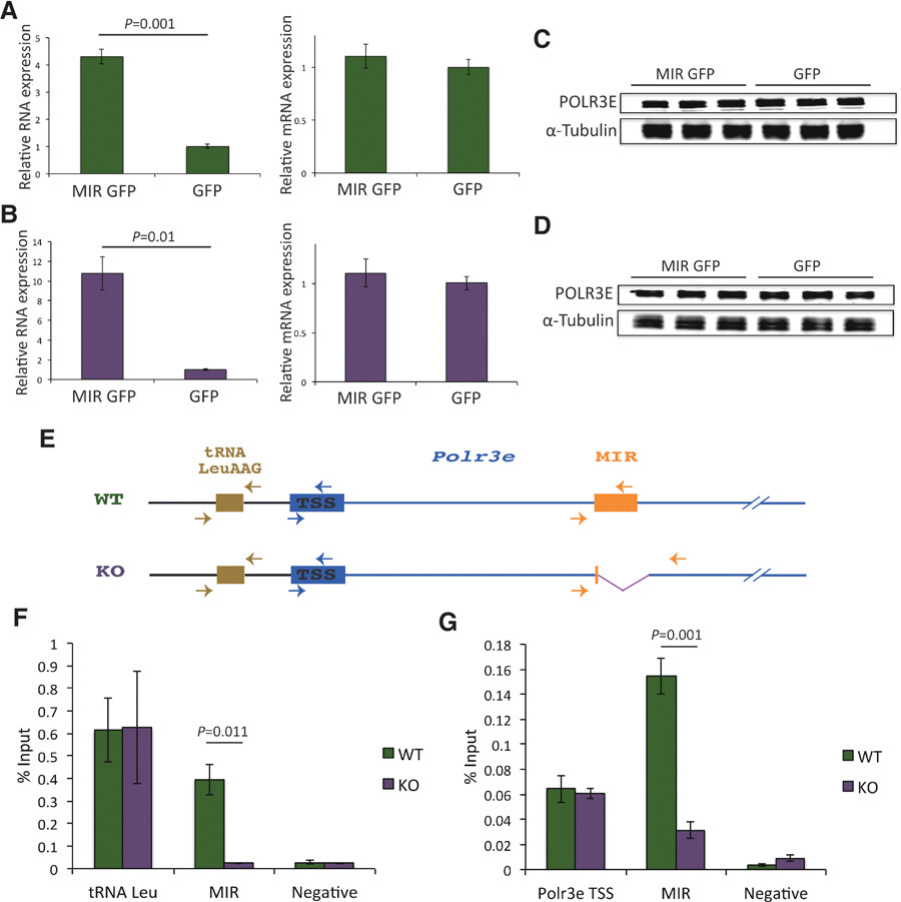
The MIR effect on *Polr3e* is mediated in *cis*. (*A*) RT-qPCR detecting the MIR (*left* panel) and total *Polr3e* mRNA (*right* panel) in wild-type ES cells transduced with a GFP lentiviral construct either containing the MIR (MIR GFP) or without the MIR (GFP), as indicated on the *X*-axis. The results were normalized to *Actb* mRNA. Error bars represent ±SEM. *n* = 3. The *P*-values were calculated using Student's *t*-test. (*B*) As in *A* but in KO11 cells. (*C*) Western blot performed with anti-POLR3E and anti-α-tubulin antibodies with protein extracts from wild-type ES cells transduced with MIR GFP or GFP lentiviral constructs. (*D*) As in *C* but with KO11 ES cell extracts. (*E*) Positions of primers used for qPCR after ChIP in wild-type and KO11 cells. (*F*) ChIP-qPCR performed with anti-RPC4 antibody. The tRNA Leu gene upstream of the *Polr3e* TSS served as a positive control. A Pol II-occupied locus (*Mycbp*) served as a negative control. The qPCR signals were normalized to input. Error bars and *P*-values are as in *A*. (*G*) ChIP-qPCR performed with anti-RPB2 antibody. The *Polr3e* TSS served as a positive control, and a Pol III-occupied locus (a tRNA Leu gene on chromosome 13) served as a negative control. The qPCR signals were normalized to the input. Error bars and *P*-values are as in *A*.

To determine whether MIR RNA is sufficient to regulate *Polr3e* expression, we overexpressed the MIR using a lentiviral vector coexpressing GFP. We did so not in only wild-type but also KO11 cells, in case the endogenous MIR levels in wild-type cells were already saturating. Although we could confirm MIR overexpression in both wild-type and KO11 ES cells by RT-qPCR (Fig. 6A,B, left panels), we did not observe any significant change in the level of total *Polr3e* mRNA (Fig. 6A,B, right panels) or POLR3E protein (Fig. 6C,D). This suggests that MIR RNA overexpressed from other loci in the genome does not affect *Polr3e* transcription or *Polr3e* mRNA translation.

The results above (Fig. 1A) revealed Pol II accumulation toward the 3′ end of the MIR, consistent with the MIR creating a roadblock to elongation. We performed ChIP-qPCR assays in mouse ES cells using antibodies against the RPC4 (POLR3D) subunit of Pol III and the RPB2 (POLR2B) subunit of Pol II. The positions of the primers used for qPCR are shown in Figure 6E and Supplemental Figure S3A. As expected, we detected a strong Pol III signal on the tRNA Leu gene upstream of the *Polr3e* TSS in both wild-type and KO11 cells, and the Pol III signal in the MIR region was detectable only in the wild-type cells (Fig. 6F; Supplemental Fig. S3B). With the anti-RPB2 antibody, we observed similar Pol II enrichment on the *Polr3e* TSS in both wild-type and MIR KO11 cells, suggesting that the presence or absence of the MIR did not affect Pol II recruitment (Fig. 6G; Supplemental Fig. S3C). Similarly, Pol II recruitment was unaffected at several locations along the *Polr3e* gene with one notable exception: The Pol II accumulation in wild-type cells just before the MIR was absent in MIR KO11 cells (Fig. 6G; Supplemental Fig. S3C). These results indicate that removal of the MIR and thus suppression of antisense Pol III transcription within the first *Polr3e* intron relieve Pol II pausing, leading to the increased expression of *Polr3e* observed above.

## Discussion

We describe a genomic arrangement, conserved in mammalian cells, in which a MIR antisense Pol III transcription unit creates a roadblock within the *Polr3e* Pol II transcription unit, leading to decreased expression of the *Polr3e* gene at both the level of mRNA and protein accumulation. The effect on protein expression was relatively modest (1.5-fold) but in line with the conclusions of a recent systematic study in yeast, where Pol II antisense transcription of ncRNAs was shown to lead, on average, to a less than twofold reduction in the protein levels encoded by the sense genes (Huber et al. 2016). However, the effect of antisense transcription was different in different conditions; in this respect, it is possible that the MIR affects *Polr3e* levels differently in different cells and tissues and under different conditions.

Insertion of the MIR into an intron placed within the EGFP-coding sequence leads to decreased EGFP expression under conditions of both low (noninduced) and high (doxycycline-induced) EGFP transcription. This is in contrast to ncRNA Pol II transcription in yeast, which seems to suppress specifically low levels of gene expression. Thus, in a genome-wide study, antisense ncRNA transcription was found to switch off corresponding sense genes under conditions of low, but not high, expression (Xu et al. 2011). Similarly, a ncRNA transcribed antisense of the GAL10 gene suppressed leaky expression of GAL10 and GAL1 in glucose-containing repressing medium but not in galactose-containing inducing medium (Lenstra et al. 2015). The inhibitory effect of the MIR under conditions of both low and high Pol II transcription may be a specificity of an antisense Pol III transcription unit as opposed to an antisense Pol II ncRNA transcription unit, or the range of the EGFP assay may not cover Pol II expression levels that might be differentially affected by MIR expression.

Our results show that the levels of the POLR3E subunit of Pol III can be regulated by the MIR. Like its yeast ortholog, Rpc37, with Rpc53, POLR3E (RPC5) forms a dimer with POLR3D (RPC4) that resembles TFIIF (Hu et al. 2002; Cramer et al. 2008). In yeast, this TFIIF-like dimer contributes to promoter opening and transcription initiation (Kassavetis et al. 2010) and is necessary, together with Rpc11, for formation of the pretermination complex and transcription termination (Arimbasseri and Maraia 2015). Mammalian POLR3E is also essential for Pol III transcription, as immunodepletion of this subunit from the Pol III complex debilitated Pol III transcription in vitro (Hu et al. 2002). Thus, the POLR3E subunit plays essential roles in the Pol III transcription process, and regulation of its levels may be critical. Although we did not observe higher levels of several Pol III products in MIR KO11 cells as compared with wild-type cells, suggesting that POLR3E was not limiting under our experimental conditions, it is likely that in other cell types or conditions in which the *Polr3e* gene is less transcribed, inhibition by the MIR, which, as mentioned above, reduces expression of lowly expressed EGFP, leads to less Pol III activity. Under such a condition, inhibition by the MIR might constitute a negative feedback loop, where overactivated Pol III would lead to increased MIR transcription and thus decreased expression of POLR3E, leading in turn to decreased Pol III activity.

How frequent is inhibition of Pol II transcription by an embedded Pol III transcription unit? A stringent analysis of Pol III occupancy in HeLa and IMR90Tert cells revealed only a handful of Pol III-occupied loci embedded within Pol II-occupied genes, but, in all of these cases, the Pol III peaks coincided with accumulations of Pol II. Thus, in these particular cultured cells, there are few potential cases. However, there is a very large number of unoccupied SINEs within Pol II transcription units: The observed tissue-specific expression of SINEs and tRNA genes (Dittmar et al. 2006; Faulkner et al. 2009) raises the possibility that some of the embedded Pol III transcription units create roadblocks for expression of their host Pol II genes in a tissue-specific manner. Moreover, there might be mechanisms other than elongation block for regulation of Pol II genes by nested Pol III transcription units.

The ineffectuality of MIR RNA overexpression to impact on *Polr3e* expression even in MIR KO11 cells, the inhibitory effect on EGFP expression of an actively transcribed MIR embedded antisense within the EGFP transcription unit, and, perhaps most telling, the accumulation of Pol II in the first *Polr3e* intron when—and only when—the MIR is present all argue for a mechanism of inhibition entailing a transcriptional interference mechanism. Transcriptional interference in overlapping genes can be modeled in several ways (Shearwin et al. 2005), one of which is disruption of transcription factor binding by the traveling polymerase. In our case, the MIR transcription unit does not overlap with the *Polr3e* promoter and must thus be interfering with Pol II elongation within the *Polr3e* gene. Interference might result from one of the Pol III transcription factors forming a roadblock to Pol II elongation. TFIIIC, which binds directly to the A and B boxes and recruits TFIIIB, has a low enrichment relative to TFIIIB subunits in ChIP-seq analyses, consistent with it detaching from the template after establishment of the Pol III transcription initiation complex (Roberts et al. 2003, 2006; Moqtaderi and Struhl 2004; Soragni and Kassavetis 2008). TFIIIB, on the other hand, has very high occupancy and is responsible, in yeast, for the inhibition by a Pol III tRNA gene of readthrough Pol II transcription from an upstream lncRNA gene, an effect that was independent on orientation (Korde et al. 2014). A TFIIIB-mediated effect might account for some of the cases that we observed in the human genome, which involve sense as well as antisense Pol III transcription units (Fig. 5), but, in the case of the MIR, the results of the EGFP assay suggest that a major roadblock function occurs only when the MIR is antisense. Thus, a more likely possibility is that, in this case, inhibition results from head-to-head collision of Pol II and Pol III. The Pol II encountering Pol III might either just slow down, pause, or have to backtrack to allow cleavage of the nascent RNA 3′ end and realignment into the catalytic site. In yeast, the collision of two Pol II machineries transcribing from convergent promoters was shown to block transcription and lead to Pol II polyubiquitylation and degradation (Hobson et al. 2012). Thus, an interesting question is whether full-length *Polr3e* mRNAs can be generated only when the MIR happens to not be transcribed by Pol III or whether Pol II, perhaps in collaboration with some bypass factors, can, at least on occasion, transcribe through the roadblock.

## Materials and methods

### Cell culture, transfection, and lentiviral transduction

V6.5 mouse ES cells were maintained on 0.1% gelatin in DMEM/F-12 GultaMAX (Gibco) supplemented with 15% ES cell-qualified fetal bovine serum (Gibco), 100 U/mL penicillin, 100 µg/mL streptomycin, 0.1 mM nonessential amino acids, 0.1 mM 2-mercaptoethanol, and 1000 U/mL LIF (Merck Millipore). Flp-In T-REx 293 cells were cultured in DMEM containing 10% tetracycline-free fetal calf serum (Bioconcept) and penicillin/streptomycin. Mouse ES cells and 293 cells were transfected with 1:4 and 1:3 (microgram:microliter) ratios of DNA to FuGENE HD transfection reagent (Promega), respectively. For production of lentiviral particles, 293FT cells were cotransfected with psPAX2, pMD2.G, and pRRLSIN.cPPT.PGK-GFP.WPRE plasmid (Addgene) containing the MIR and ∼140 bp of 5′ and 3′ flanking genomic sequence (the same sequence used for EGFP assay). The supernatant of transfected cells was collected 48 and 72 h after transfection, and the lentiviral particles were concentrated by ultracentrifugation. Transduced ES cells were selected by FACS.

### CRISPR/Cas9 genome engineering

CRISPR/Cas9 genome engineering was performed as described (Ran et al. 2013). To delete the MIR genomic sequence, preserving its 3′ end region, we used two short guide RNAs (sgRNAs) (see Supplemental Fig. S4A). After cotransfection of mouse ES cells with pSpCas9(BB)-2A-GFP vectors containing sgRNA1 and sgRNA2 and selection by FACS, we screened for MIR deletions by PCR on genomic DNA of single-cell-derived ES cells with a pair of primers flanking the MIR (Supplemental Fig. S4B) and by sequencing of some of the PCR products (Supplemental Fig. S4C). We obtained different deletion lengths (Supplemental Fig. S4D).

### RNA extraction and RT-qPCR

Total RNA was extracted and DNase I-treated with the miRNeasy minikit (Qiagen). One microgram of RNA was reverse-transcribed with M-MLV reverse transcriptase (Promega) with either gene-specific primers or random hexamers. The sequences of the qPCR primers are listed in Supplemental Table S2.

### Western blot, Northern blot, and in vitro transcription

The primary antibodies for Western blots (anti-POLR3E [RPC5], CS1542 [Hu et al. 2002], and anti-γ- and α-Tubulin [Santa Cruz Biotechnology]) were used at 1:1000 dilutions. For Northern blots, RNA was extracted with TRIzol reagent (Ambion) according to the manufacturer's instructions, and 20 µg (Fig. 3D) or 10 µg (Supplemental Fig. S2) of total RNA was used. The oligonucleotide probe sequences are listed in Supplemental Table S2. In vitro transcription was performed according to Lobo et al. (1992).

### ChIPs

ChIPs were performed as described in Orioli et al. (2016). Chromatin was sheared with a Bioruptor sonicator (Diagenode). Sonicated chromatin from 5 million cells was used for each ChIP. The antibodies for immunoprecipitation were anti-RPB2 (POLR2B) (Santa Cruz Biotechnology, H-201) and anti-RPC4 (POLR3D) (Canella et al. 2012). The sequences of qPCR primers used after ChIP are listed in Supplemental Table S2.

## Supplementary Material

Supplemental Material
