# Supplementary material for: Transcriptional interference by RNA polymerase III affects expression of the Polr3e gene
Source: Genes Dev. 2017 Feb 15;31(4):413–21. doi: 10.1101/gad.293324.116 (PMC5358760; doi:10.1101/gad.293324.116)
Supplement: Supplemental Material [file supp_31_4_413__index.html]

Transcriptional interference by RNA polymerase III affects expression of the Polr3e gene — Supplemental Material 

# Transcriptional interference by RNA polymerase III affects expression of the *Polr3e* gene

## Supplemental Material

undefined

- Supplemental\_Table\_S1.xlsx
- Supplemental\_Data.pdf
