# Supplementary material for: Transcriptional interference by RNA polymerase III affects expression of the Polr3e gene
Source: Genes Dev. 2017 Feb 15;31(4):413–21. doi: 10.1101/gad.293324.116 (PMC5358760; doi:10.1101/gad.293324.116)
Supplement: Supplemental Material [file supp_gad.293324.116_Supplemental_Data.pdf]

Supplemental Figure Legends

Figure S1. Alignment of the MIR-like sequence in the first intron of the *Polr3e* gene in different mammalian species. The alignment was performed with ClustalW with the default settings for nucleotide sequences; the graphic representation was generated with Jalview. The A and B boxes are indicated.

Figure S2. Northern blot for Pol III transcribed 5S rRNA, U6 snRNA, and pre-tRNA Ile in MIR KO and WT ES cells. The Pol II-transcribed U3 snoRNA was used as an internal control for RNA loading.

Figure S3. Pol III and Pol II occupancy sampling over the *Polr3e* gene. **A)** Position of primers used for qPCR after ChIP. The exon-intron structure of the *Polr3e* gene is redrawn from the Ensembl genome browser. **B)** ChIP-qPCR performed with an anti-RPC4 antibody. **C)** ChIP-qPCR performed with an anti-RPB2 antibody.

Figure S4. CRISPR/Cas9-mediated deletion of the MIR in mouse ES cells. **A)** sgRNA design for deletion of the MIR. The MIR sequence, the A and B boxes, and the target sequences are shown. The PAM sequences are shown in bold, and the predicted double strand break sites are indicated by double arrows. **B)** Products of PCRs performed on genomic DNA from several clones with a pair of primers flanking the MIR, separated on an agarose gel. **C)** Characterization of the deletions in different MIR KO clones. The sequencing results of the PCR products obtained from several clones are shown aligned to the WT sequence. The A (yellow) and B (green) boxes are highlighted; the MIR sequence is shown in bold. **D)** Schematic view of the

deletions in the different clones. The MIR A (yellow) and B (green) boxes, TSS (arrow), and termination site (end of black line) are shown.

Supplemental Tables

Table S1. List of ENCODE Pol III peaks located inside human Pol II genes. The table lists all ENCODE RPC1 peaks located inside Pol II transcription units. Column A gives links to a UCSC genome browser session showing RPC4, RPC1, ENCODE RPC1, Pol II, DSIF, and NELF tracks. The pol II, DSIF, and NELF tracks are from Liu et al., 2014. Column D indicates the direction of the Pol III transcription unit relative to the genome. Column G refers to scores (columns R, S, and T) calculated as in Canella et al., 2012, for the regions indicated (columns P and Q), for anti-RPC4 ChIP-seq data obtained from IMR90Tert cells under serum replete conditions (Orioli et al. 2016). Column H refers to scores (columns U, V, W) calculated as in Canella et al., 2012, for the regions indicated (columns P and Q), for the ENCODE anti-RPC1 ChIP-seq data obtained from HeLa cells. Column I refers to scores (column X), calculated as in ENCODE, for the regions indicated (columns P and Q). Y stands for yes, N for no. Column L indicates the direction of the Pol II gene relative to the genome.

Table S2. Sequences of oligonucleotides used in RT-qPCR, ChIP-qPCR, and northern blots.

Yeganeh_Fig S1

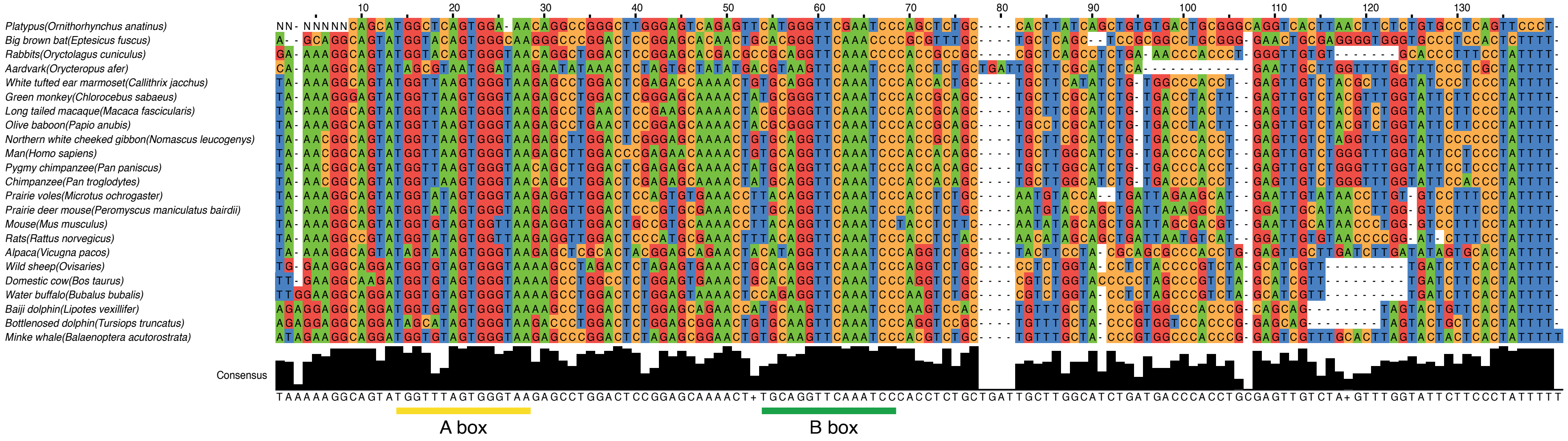

Yeganeh_Fig S2

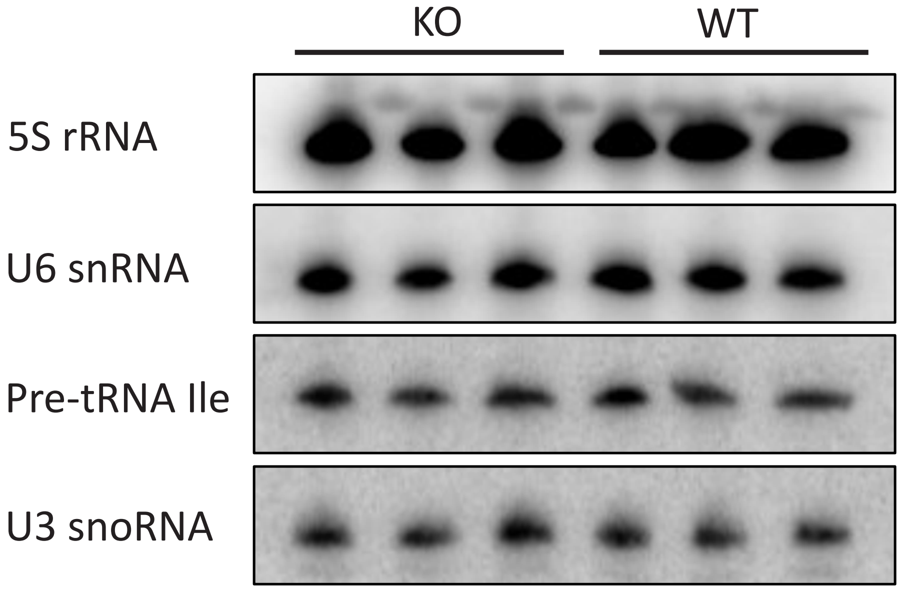

A

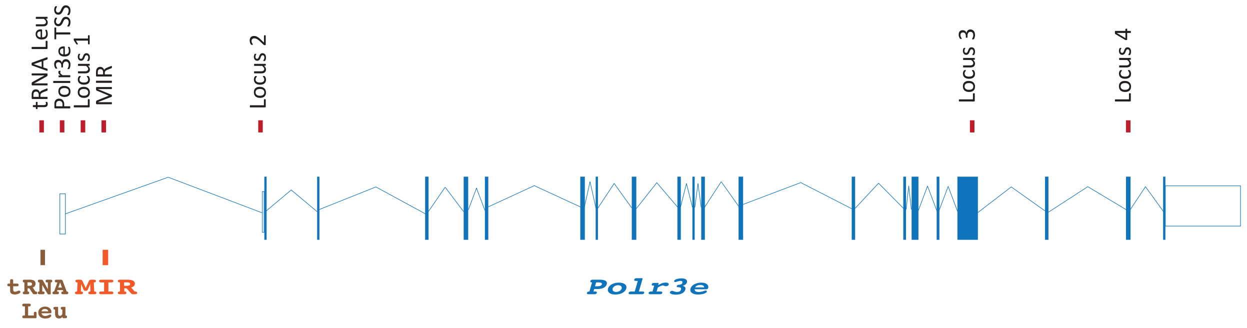
tRNA Leu
Polr3e TSS
Locus 1
MIR
Locus 2
Locus 3
Locus 4
tRNA Leu
MIR
Polr3e

B

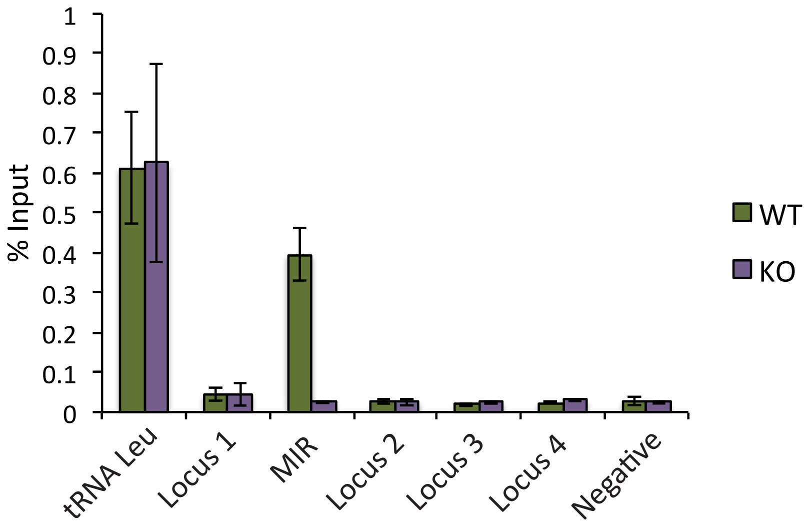
% Input
1
0.9
0.8
0.7
0.6
0.5
0.4
0.3
0.2
0.1
0
tRNA Leu
Locus 1
MIR
Locus 2
Locus 3
Locus 4
Negative
WT
KO

C

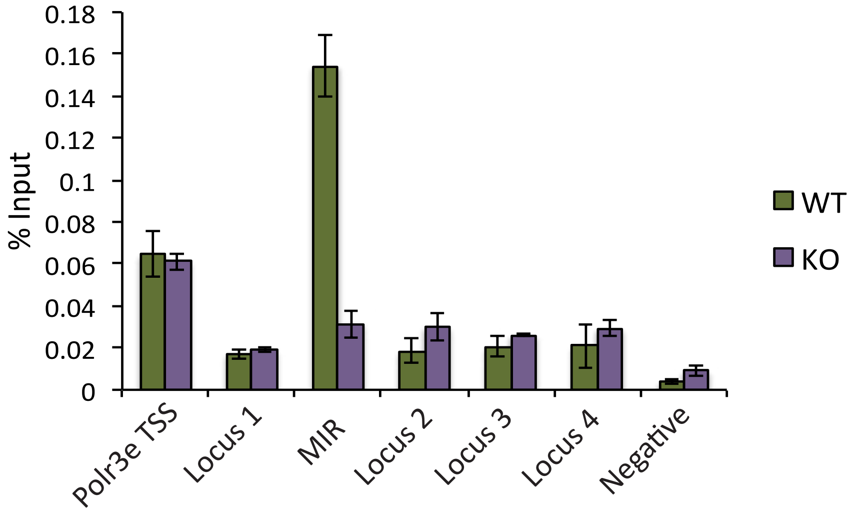
% Input
0.18
0.16
0.14
0.12
0.1
0.08
0.06
0.04
0.02
0
Polr3e TSS
Locus 1
MIR
Locus 2
Locus 3
Locus 4
Negative
WT
KO

Yeganeh_Fig S4

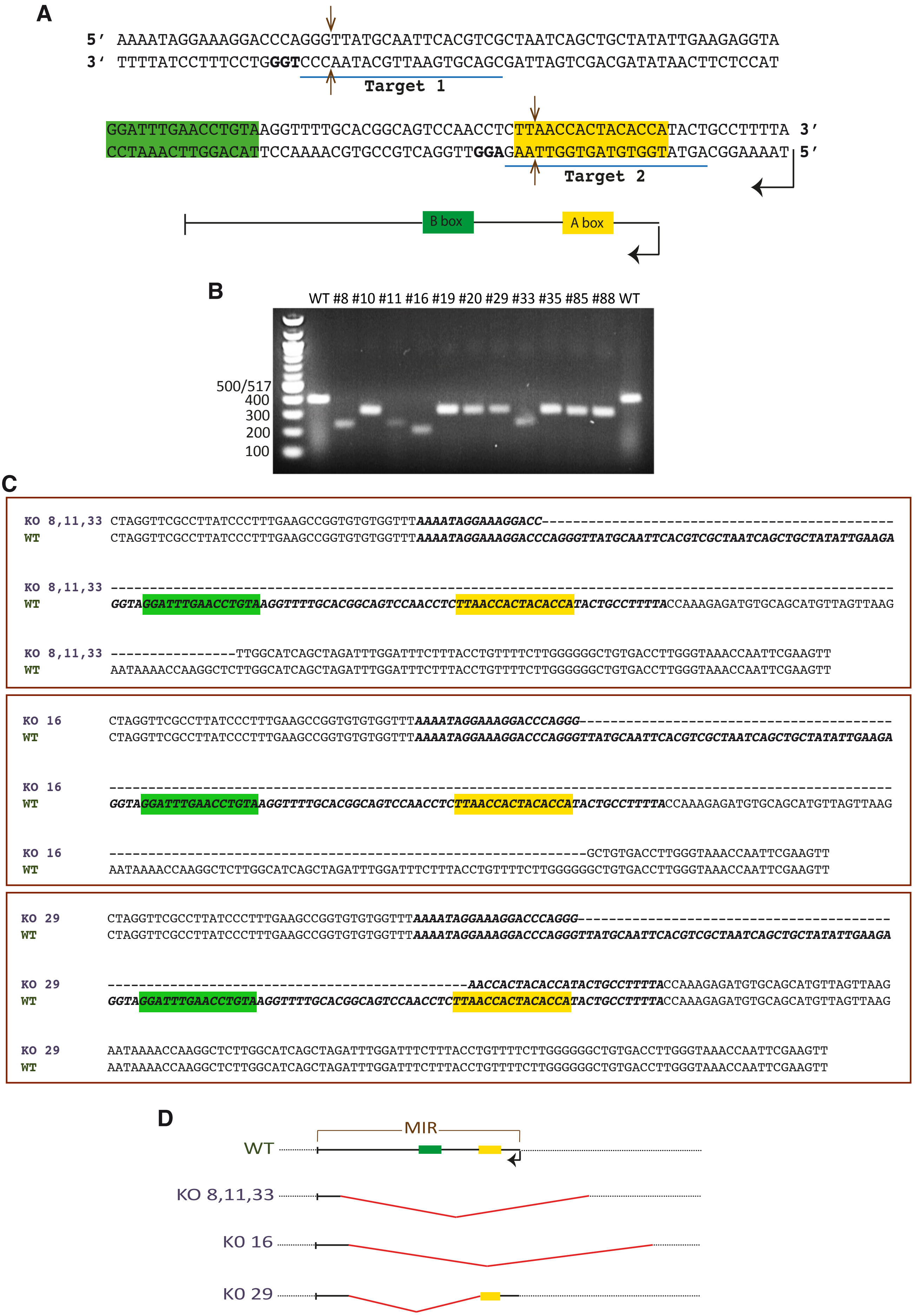

RT-qPCR	
Oligonucleotide name	Sequence (5' to 3')
MIR- Fwd	GACTGCCGTGCAAAACCTTA
MIR- Rev	TTATGCAATTCACGTCGCTAA
Total *Polr3e*- Fwd	AGAGACAGTTTGTGCTCACG
Total *Polr3e*- Rev	TGAGACGCCACTGAAGAGTA
Pre-*Polr3e*-Fwd	CTGTTGGCTGCTACTGAACA
Pre-*Polr3e*-Rev	CTCTTCGCAGTGCTTTGACT
Mature *Polr3e*- Fwd	CACATATTCCTCAAAGCTGATGG
Mature *Polr3e*- Rev	GATGCCATGTAAAGGTGTCAGG
Actb- Fwd	CTAAGGCCAACCGTGAAAAGAT
Actb- Rev	CACAGCCTGGATGGCTACGT
Gapdh- Fwd	AGGTCGGTGTGAACGGATTTG
Gapdh- Rev	TGTAGACCATGTAGTTGAGGTCA

Northern blot	
Oligonucleotide name	Sequence (5' to 3')
MIR	TTATGCAATTCACGTCGCTAA
U87 snoRNA	TCACACCCATGACTGCCACT
5S rRNA	TTAGCTTCCGAGATCAGACG
U6 snRNA	CACGAATTTGCGTGTCATCC
Pre-tRNA Ile	ATCGCTTACGCCTAGCACTG
U3 snoRNA	GGAGGGAAGAACGATCATCA

ChIP-qPCR	
Oligonucleotide name	Sequence (5' to 3')
MIR- Fwd	TTCGCCTTATCCCTTTGAAG
MIR- Rev (WT)	CAGTATGGTGTAGTGGTTAAG
MIR- Rev (KO11)	TACTCTTCTCATTAGCTGTGC
chr7-tRNA Leu- Fwd	TTAGAAAACGACGTCAACAGC
chr7-tRNA Leu- Rev	GACAAAAGAAAAAGCCTGCCT
Polr3e TSS- Fwd	CATTGTGGGTAAGAGGAAGC
Polr3e TSS- Rev	TATCAGGCAGCGGCCATGTTC
Mycbp- Fwd	ACTCGAAGCGCGAGCAGT
Mycbp- Rev	CTCACCTTTCGTCAGCGTGT
chr13- tRNA Leu- Fwd	AGGTTCACGGAAGGTCTG
chr13- tRNA Leu- Rev	CTATGGCTTCCTCGCTCTG
Locus 1- Fwd	GCTTTCGGAAGAGTGGGAAG
Locus 1- Rev	AGAGTTGACCAGGTTCAACG
Locus 2- Fwd	CACAGACACAGAAAGGAGACC
Locus 2- Rev	ACCTCACAGTCCTCAACTCG
Locus 3- Fwd	AGAGACAGTTTGTGCTCACG
Locus 3- Rev	TGAGACGCCACTGAAGAGTA
Locus 4- Fwd	CATCGGCAGGTTTTGCTTG
Locus 4- Rev	CTTTAGCACTTTATCCACCTCC
